# Vδ2 T-Cells Kill ZIKV-Infected Cells by NKG2D-Mediated Cytotoxicity

**DOI:** 10.3390/microorganisms7090350

**Published:** 2019-09-12

**Authors:** Eleonora Cimini, Alessandra Sacchi, Sara De Minicis, Veronica Bordoni, Rita Casetti, Germana Grassi, Francesca Colavita, Concetta Castilletti, Maria Rosaria Capobianchi, Giuseppe Ippolito, Maria Giovanna Desimio, Margherita Doria, Chiara Agrati

**Affiliations:** 1 Immunology and Pharmacology Laboratory, National Institute for Infectious Diseases Lazzaro Spallanzani-IRCCS, via Portuense 292, 00149 Rome, Italy; eleonora.cimini@inmi.it (E.C.); alessandra.sacchi@inmi.it (A.S.); demsara@hotmail.it (S.D.M.); veronica.bordoni@inmi.it (V.B.); rita.casetti@inmi.it (R.C.); germana.grassi@inmi.it (G.G.); 2 Virology Laboratory, National Institute for Infectious Diseases Lazzaro Spallanzani-IRCCS, via Portuense 292, 00149 Rome, Italy; francesca.colavita@inmi.it (F.C.); concetta.castilletti@inmi.it (C.C.); maria.capobianchi@inmi.it (M.R.C.); 3 Scientific Direction; National Institute for Infectious Diseases Lazzaro Spallanzani-IRCCS, via Portuense 292, 00149 Rome, Italy; giuseppe.ippolito@inmi.it; 4 Division of Immunology and Infectious Diseases, Academic Department of Pediatrics, Bambino Gesù Children’s Hospital, IRCCS, Piazza Sant’Onofrio 4, 00165 Rome, Italy; mariagiovanna.dsm@gmail.com (M.G.D.); doria@uniroma2.it (M.D.)

**Keywords:** ZIKV, innate immunity, Vδ2 T-cells, antiviral activity, NKG2D, cytotoxicity, perforin

## Abstract

An expansion of effector/activated Vδ2 T-cells was recently described in acute Zika virus (ZIKV)-infected patients, but their role in the protective immune response was not clarified. The aim of this study was to define the antiviral activity of Vδ2 T-cells against ZIKV-infected cells. The Vδ2 T-cells expansion and their cytotoxic activity against ZIKV-infected cells were tested *in vitro* and analyzed by RT-PCR and flow cytometry. We found that ZIKV infection was able to induce Vδ2 T-cells expansion and sensitized A549 cells to Vδ2-mediated killing. Indeed, expanded Vδ2 T-cells killed ZIKV-infected cells through degranulation and perforin release. Moreover, ZIKV infection was able to increase the expression on A549 cells of NKG2D ligands (NKG2DLs), namely MICA, MICB, and ULBP2, at both the mRNA and protein levels, suggesting the possible involvement of these molecules in the recognition by NKG2D-expressing Vδ2 T-cells. Indeed, the killing of ZIKV-infected cells by expanded Vδ2 T-cells was mediated by NKG2D/NKG2DL interaction as NKG2D neutralization abrogated Vδ2 cytotoxicity. Our data showed a strong antiviral activity of Vδ2 T-cells against ZIKV-infected cells, suggesting their involvement in the protective immune response. Other studies are necessary to investigate whether the lack of Vδ2 T-cells expansion *in vivo* may be associated with disease complications.

## 1. Introduction

Zika virus (ZIKV) is an arbovirus belonging to the Flaviviridae family, genus Flavivirus [1]. ZIKV was introduced in Brazil between 2013 and 2014, spread rapidly within the northeast part of the country, and was repeatedly introduced into various regions of the Americas [2]. The clinical syndrome caused by ZIKV in humans consists in a mild influenza-like illness resolving within days that occurs in approximately 20% of infected individuals [3]. The most common signs and symptoms of ZIKV infection arise after 3–7 days from mosquito bite and include fever (72%), arthralgia and myalgia (65%), conjunctivitis (63%), headache (46%), fatigue and/or rash [4]. In the last outbreak, ZIKV has emerged as a global health problem; in fact, ZIKV generated an explosive epidemic causing Guillain–Barré syndrome in adults [5,6,7] and congenital diseases during pregnancy [8,9]. Given the range of clinical symptoms, there is a pressing need to elucidate the protective immunological profile and to identify the immune signature associated with complication. Effective control of ZIKV involves both humoral and cell-mediated immune responses. Neutralizing antibodies are produced after ZIKV infection, control viral replication [10], and correlate with protection against secondary infection [11,12,13]. 

At present, various studies aimed to understand T-cell responses against ZIKV have been reported. In mice, polyfunctional cytotoxic CD8+ T-cells are activated [14] and reduce ZIKV burden, whereas their depletion resulted in greater ZIKV infection and mortality [15]. Adoptive transfer of ZIKV-specific CD8+ T-cells can protect against ZIKV infection [16], confirming the role of CD8+ T-cells against ZIKV. Nevertheless, CD8+ T-cells could also have pathological consequences in the brain [17] resulting in ZIKV-associated paralysis [18]. The role of CD4+ T-cells in the protection/pathogenesis of ZIKV infection is not fully elucidated. CD4+ T-cells proliferated and produced Th1 cytokines in mice [19], and their depletion reduced humoral response [20]. In ZIKV-infected humans, CD4+ T-cells differentiated into effectors cells but showed an impaired IFN-γ production [21]. 

Among cells of the innate immunity, Vδ2 T-cells are known to contribute to the antimicrobial immune response [22]. They are activated both *in vitro* and *in vivo* by using phosphoantigens (PhAgs) [23] without any MHC restriction and are able to produce pro-inflammatory cytokines [24].

Vδ2 T-cells can also display a potent MHC unrestricted cytotoxic activity against tumors and infected cells through the engagement of their NK receptor group 2 member D (NKG2D) receptor [25,26]. NKG2D is expressed by the majority of γδ T-cells, as well as by NK, CD8+ T-cells and by subsets of NKT cells and CD4+ T-cells, and recognizes several ligands (NKG2DLs): the major histocompatibility complex I-related chain A and B proteins (MICA and MICB) and UL16 binding protein 1–6 (ULBP1–6) [27]. Expression of NKG2DLs is highly restricted in normal tissues but can be induced during viral infection and tumor transformation, eliciting recognition and elimination of virus-infected cells and tumors by NKG2D+ immune cells. The role of Vδ2 T-cells during Flavivirus infection is not clearly depicted. We demonstrated that Vδ2 T-cells are able to perform a cytolitic activity against WNV (West Nile Virus) by releasing perforin. Indeed, they can also produce cytokines with antiviral activity [28]. In acute Dengue (DENV) infection, Vδ2 T-cells are able to exert a potent antiviral activity by expressing CD107a and by producing IFN-γ against DENV-infected cells [29]. During acute ZIKV infection in humans, an expansion of Vδ2 T-cells was observed. These expanded Vδ2 T-cells showed an effector profile, were enriched of Granzyme B and were able to produce IFN-γ when stimulated with a specific antigen [21]. Nevertheless, their involvement in the anti-ZIKV immune response has not been demonstrated. The aim of this work was to investigate the antiviral capability of Vδ2 T-cells against ZIKV infection.

## 2. Materials and Methods 

### 2.1. A549 Maintenance

A549 cells were grown in Dulbecco’s modified eagle medium (DMEM) supplemented with Fetal Bovin Serum 10%, 2 mmol/L L-Glutamine, 50 IU/mL Penicillin and 50 μg/mL Streptomycin (EuroClone, Siziano, Italy) in a humidified incubator at 37 °C with 5% of CO_2_. Passaging of the cells was carried out twice a week, reaching a maximum density of 80–90%.

### 2.2. ZIKV Infection

A549 cells were infected with the ZIKV strain MR766 (UVE/ZIKV/1947/UG/MR766 available on http://www.european-virus-archive.com/as EVAg no. 001v-EVA143).

A549 cells, plated in complete DMEM medium (70.000 cells/250 µL/well) in a 48-well plate the day before, were infected with ZIKV at MOI 1 (multiplicity of infection) for 2 h in serum-free medium at 37 °C and 5% of CO_2_. After 48 h, cells were washed with PBS 1X, and co-cultured with Peripheral Blood Mononuclear Cells (PBMC) or expanded Vδ2 T-cells. Non-infected A549 cells (mock) were used as control in all the experiments. 

### 2.3. Lymphocytes Isolation

PBMC were obtained from healthy donors (HD) by gradient centrifugation (Lympholyte, cat. #CL5020, Cederlane, Ontario, Canada), counted by Trypan blue exclusion, and suspended (1× 10^6^ cells/mL) in culture medium (RPMI-1640 supplemented with 10% Fetal Bovine Serum, 2 mM L-glutamine, 50 IU/mL Penicillin and 50 μg/mL Streptomycin, EuroClone, Siziano, Italy). 

Expanded Vδ2 T-cells were obtained by culturing PBMC of HD with a Phosphoantigen (IPH1101, 3 µM; Innate Pharma, Marseille, France) plus IL-2 (100 IU/mL) at 37 °C and 5% of CO_2_. Culture medium plus IL-2 (100 IU/mL) was added at day 7. After 12 days, cells were isolated by magnetic separation (Miltenyi Biotec, Bologna, Italy) obtaining a purity of >95%. Vδ2 T-cells frequency were analyzed by flow cytometry before and after culture and were used for the experiments of this study. 

### 2.4. Real-time qPCR

Total RNA was extracted with TRIzol (Life Technologies, Monza, Italy) from A549 cells uninfected and infected with ZIKA. Aliquots of total RNA were used to generate cDNA using random hexamers and the resulting cDNA (25 ng) was amplified in triplicate using the SensiFAST SYBR Green PCR master mix (all from Bioline, Rome, Italy). For each target (MICA, MICB, ULBP1, ULBP2, and the G6PDH rRNA calibrator gene), forward (F) and reverse (R) primers were designed across exons to avoid amplification of genomic DNA, as validated in pilot assays: 

MICA(F), 5′GGCAGAAGATGTCCTGGGAAA-3′; 

MICA(R), 5′-GAGGGAATGCAAGCCTTCTTTC-3′;

MICB(F), 5′-TTCCATATGTTTCTGCTGCTATG3′; 

MICB(R), 5′-CTCACAAGCTCTGGACCCTC-3′; 

ULBP1(F), 5′-GGCCGCCAGCCCCGCGTTC-3′; 

ULBP1(R), 5′- GACAGTGTGTGTCGACCCATCC-3′; 

ULBP2(F), 5′-TTACACACCCAAGGAACCCCT-3′; 

ULBP2(R), 5′-TCGCCATGTCCTCAGGCAC-3′; 

G6PDH(F), 5′-ATCGACCACTACCTGGGCAA-3′; 

G6PDH(R), 5′-TTCTGCATCACGTCCCGGA-3′. 

The cycling conditions were 95 °C for 2 min, followed by 40 cycles of 95 °C for 5 s and 60 °C for 30 s. Real-time PCR was performed using Applied Biosystems StepOne plus (Life Technologies, Monza, Italy). 

### 2.5. Flow Cytometry and Antibodies 

The following antibodies were used to analyze Vδ2 T-cells: anti-Vδ2 FITC, anti-CD3 PerCP Cy5.5 and anti-CD3 Pacific Blue (all BD Biosciences, Milan, Italy), anti-NKG2D APC (Beckman Coulter, Milan, Italy) or APC isotype control Ab (Mouse IgG1 clone; BD Pharmingen, San José, CA). Briefly, cells were stained with specific monoclonal antibodies for 20 min at 4 °C, fixed in 1% of Paraformaldehyde (PFA, Sigma, Rome, Italy) for 5 min at room temperature, and acquired on a flow cytometer (FACS Canto II, Becton Dickinson, Milan, Italy). 

The ability of ZIKV to induce Vδ2 T-cells degranulation was monitored by adding CD107a mAb (1:200; anti-CD107a APC; BD Biosciences, Milan, Italy) in culture and its expression was analyzed by flow cytometry. 

ZIKV+ cells and NKG2DL (anti-MICA/B PE, anti-ULBP-1 PE, anti-ULBP-2 PE; R&D System, Minnesota, USA) expression were analyzed by flow cytometry. Briefly, cells were stained with a specific cocktail of NKG2DL monoclonal antibodies for 20 min at 4 °C, fixed in 1% of Paraformaldehyde (PFA, Sigma, Rome, Italy) for 5 min at room temperature. After fixation, cells were washed once with buffer and stained with anti-Panflavi primary antibody (mouse anti-flavivirus group antigen monoclonal antibody, clone D1-4G2-4-15; Millipore, Milan, Italy) [30] for 20 min at RT in permeabilizing solution (PBS 0.1% NaN3, 1% BSA, 0.5% saponin). Isotype control primary antibody (Mouse IgG2a Neg control, MABC004F, Millipore, Milan, Italy) was used as control. After one wash cells were stained with a secondary antibody [(Fluorescein isothiocyanate (Fitc) conjugated affinity purified secondary antibody, clone Gt x Ms IgG Fluor, AP181F (Millipore, Milan, Italy) or Allophycocyanin (APC) labeled goat anti-mouse Ig, polyclonal, category 550826, BD Pharmingen, San Josè, CA)] for 20 min at RT in permeabilizing solution, and acquired by flow cytometry. Data were analyzed with FACS DIVA software. 

### 2.6. Cytotoxic Assay

To evaluate the cytotoxic capability of Vδ2 T-cells against ZIKV+ infected cells, overnight co-cultures of expanded Vδ2 T-cells with mock/ZIKV+ cells at ratio 1:1 and 5:1 were performed. After 18 h, mock and ZIKV-infected cells apoptosis/necrosis was evaluated by using AnnexinV kit (Annexin V-FITC Kit; eBiosciences, Bender MedSystems, Wien, Austria). Briefly, cells were labeled with anti-CD3-PB for 20 min at 4 °C, washed and labeled with Annexin V-FITC for 10 min at RT. After one wash, cells were labeled with PI (20 µg/mL) and immediately acquired by flow cytometry. A549 apoptosis was evaluated by gating on CD3 negative target cells. In all experiments, the spontaneous A549 apoptosis/necrosis (the percentage of mock/ZIKV+ apoptotic/necrotic cells without expanded Vδ2 T-cells) was subtracted to all conditions. 

### 2.7. Perforin and Cytokines Quantification 

To quantify Perforin, Interferon-γ (IFN-γ), and Tumor Necrosis Factor-α (TNF-α) released by Vδ2 T-cells, we co-cultured expanded Vδ2 T-cells with mock/ ZIKV-infected cells. After 18 h, supernatants were collected and Perforin/IFN-γ/TNF-α release was analyzed with an anti-Perforin, anti-Human IFN-γ and anti-Human TNF-α ELISA Kits (Biovendor, Milan, Italy; Thermo Scientific and Enzo Lifesciences, Rome, Italy; respectively), according to manufacturers’ instructions. Data of ELISA tests were subtracted of spontaneous release. 

### 2.8. NKG2D Blocking Assay 

Expanded Vδ2 T-cells were pre-treated with anti-NKG2D blocking antibody (1 µg/1 ×10^6^ cells; Monoclonal Mouse IgG1 Clone: 149810, R&D System, Minnesota, USA) in PBS at RT for 10 min. After incubation, cells were washed twice with PBS, suspended in complete medium and cultured with mock/ZIKV-infected cells, at 1:1 ratio. 

### 2.9. Statistical Analysis 

GraphPad Prism software (version 7.00 for Windows Professional) was used to perform statistical analysis and graphs. Non-parametric Wilxocon test was used to compare data. *p* value < 0.05 were considered statistically significant (* *p* <  0.05, ** *p* < 0.01, *** *p* < 0.001).

## 3. Results

### 3.1. ZIKV Replication in A549 Cells

**Figure 1:**
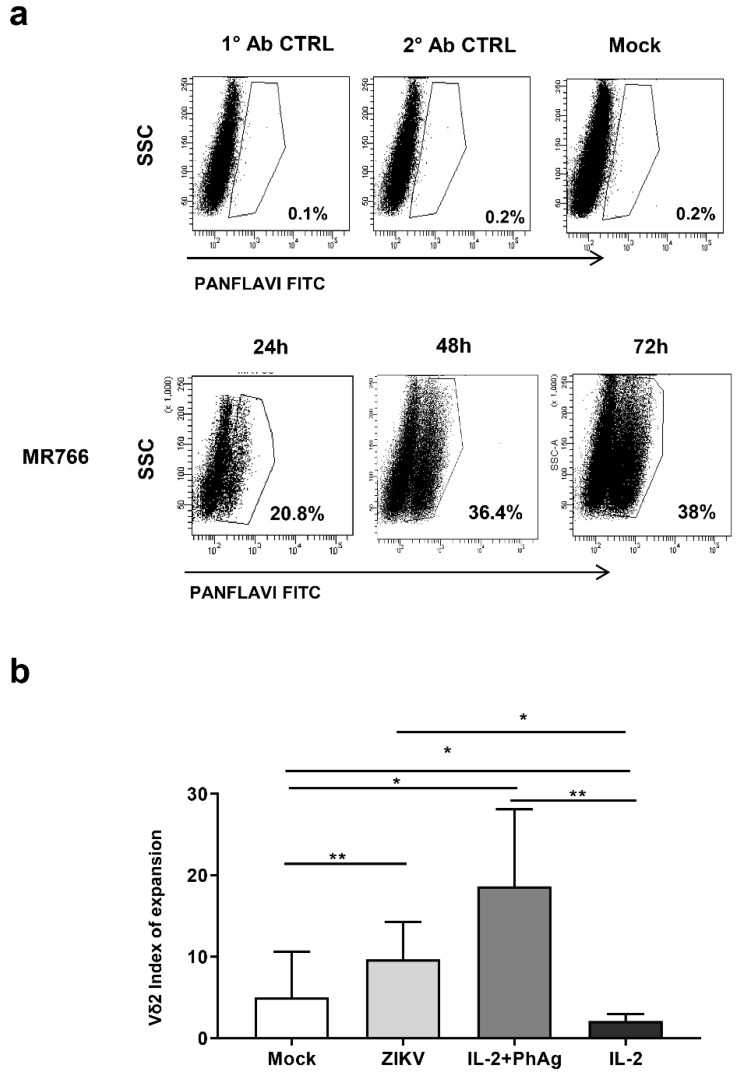
(**a**) Zika virus (ZIKV) infects A549 cells and elicits expansion of Vδ2 T-cells. A549 cells were infected with ZIKV MR766 strain and the frequency of ZIKV+ cells was analyzed by flow cytometry using the PANFLAVI antibody after 24, 48, and 72 h. Flow cytometry panels of one representative experiment out of 15 are shown. PANFLAVI+ gate was set to include ≤0.2% of events when using primary or secondary antibody isotype controls or uninfected cells (mock). (**b**) Expansion index of Vδ2 T-cells in co-culture with ZIKV-infected cells was shown. A549 cells were not infected (mock) or infected with ZIKV MR766 and cultured with peripheral blood mononuclear cells (PBMC) of healthy donors (HD). IL-2 or IL-2 + PhAg were added in parallel cultures as controls. Vδ2 T-cells frequency was calculated at baseline (T0) and after 7 days (T7). The expansion index was calculated by dividing Vδ2 T-cells frequency at 7 days in respect to T0. Results from 13 independent experiments are shown. Data were represented as median + interquartile range. * *p* < 0.05; ** *p* < 0.01.

To study the involvement of Vδ2 T-cells in the anti-ZIKV response, we set up an *in vitro* model of ZIKV-replication in a permissive tumor cell line (A549 cells) using one ZIKV strain: MR766 African strain. The frequency of ZIKV-infected cells (ZIKV +) was monitored at 24 h, 48 h and 72 h post-infection by flow cytometry [28]. We found that ZIKV MR766, the African well-adapted virus, was able to efficiently replicate in A549 cells as shown by the increase of ZIKV+ overtime (Figure 1A). Therefore, we decided to perform our experiments using A549 cells after 48 h of ZIKV MR766 infection. In this condition, we obtained the higher frequency of ZIKV-infected A549 cells able to be cultured for further days without confluence-induced cell death. 

### 3.2. Vδ2 T-cells Recognize and Kill ZIKV-infected Cells 

During acute ZIKV infection in humans, an expansion of Vδ2 T-cells has been reported [21]. In order to define the capability of ZIKV-infected cells to expand Vδ2 T-cells *in vitro*, we co-cultured mock or ZIKV-infected A549 cells with PBMC of HD for 7 days in the presence of IL-2. As a positive control of Vδ2 T-cells expansion, PBMC were also stimulated with either PhAg and IL-2 or IL-2 alone. The expansion was evaluated by using a proliferation index defined as the ratio between the frequency of Vδ2 +CD3 + cells before (T0) and after 7 days (T7) of co-culture (T7/T0). As reported previously, we found that mock-A549 induced Vδ2 T-cell proliferation (Figure 1B) [21]. Interestingly, ZIKV-infected A549 cells significantly increased the expansion of Vδ2 T-cells above the levels observed with mock A549 cells (*p* < 0.001) (Figure 1B), indicating that ZIKV infection enhanced Vδ2 T-cells expansion.

**Figure 2:**
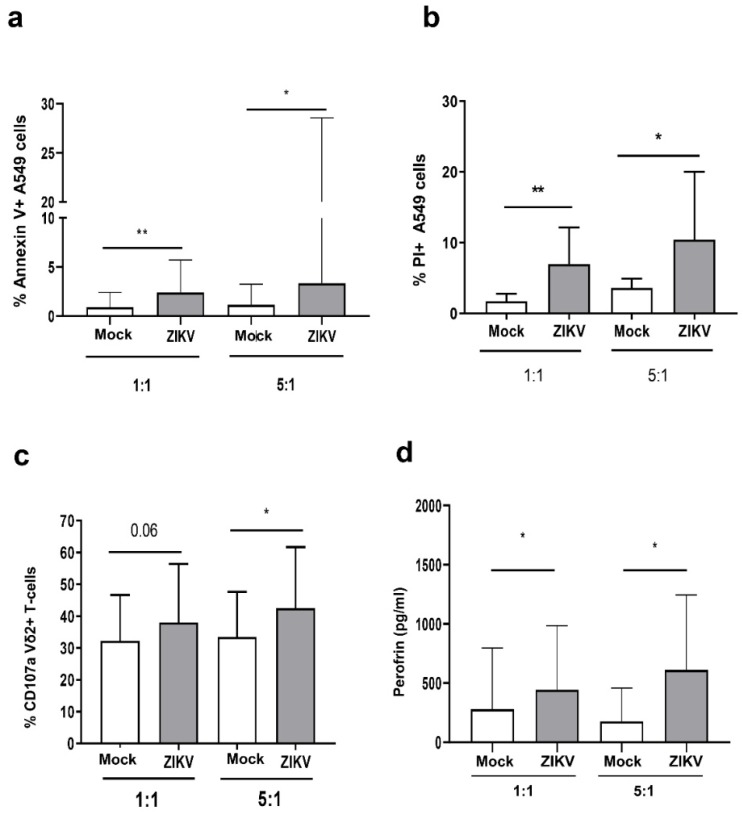
ZIKV sensitized A549 cells to Vδ2 T-cell-mediated killing. (**a**–**c**) Expanded Vδ2 T-cells were cultured overnight with mock (white bars) or ZIKV-infected A549 cells (grey bars) cells, at 1:1 and 5:1 ratios. (**a**,**b**) After 18 h, apoptotic/necrotic A549 cells were analyzed by measuring AnnexinV and PI expression by flow cytometry (*n* = 15). (**c**) The frequency of Vδ2 + /CD107a+ cells was measured after the culture with mock/ZIKV-infected cells (*n* = 7). (**d**) Perforin quantification in the supernatants of the same experiments was shown. In all experiments, background (mock/ZIKV+ apoptotic/necrotic cells without expanded Vδ2 T-cells) was subtracted to all conditions. Data were represented as median + interquartile range. * *p* <0.05; ** *p* < 0.01.

**Figure 3:**
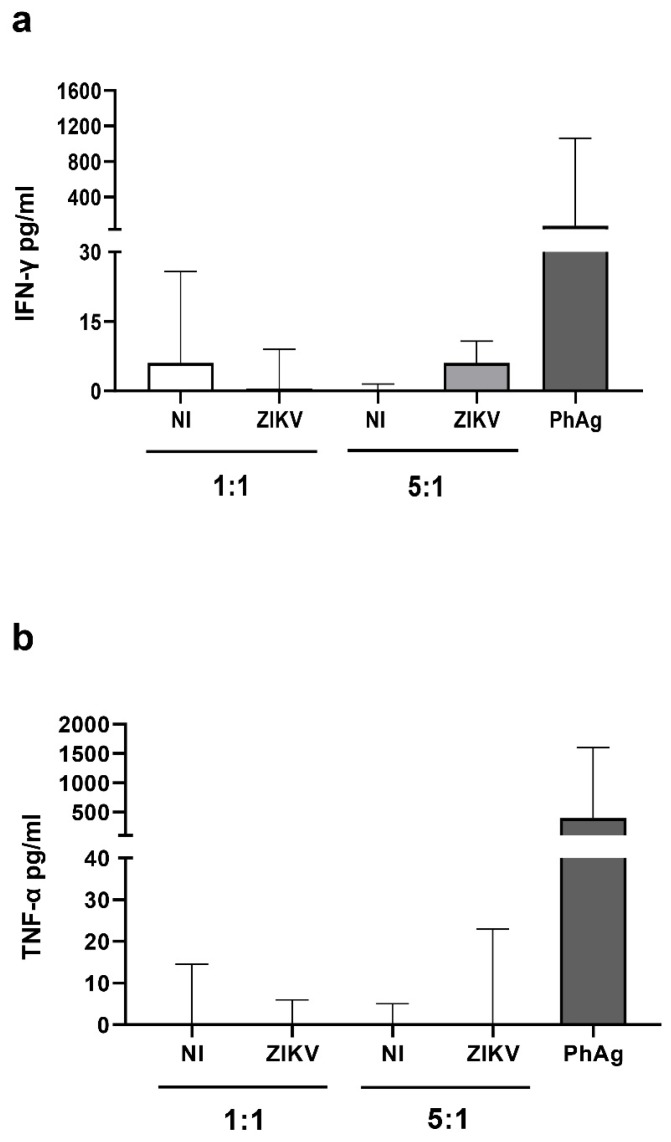
ZIKV-infected A549 cells did not induce IFN-γ and TNF-α production by expanded Vδ2 T-cells. (**a**,**b**) Expanded Vδ2 T-cells were cultured overnight with mock (white bars) or ZIKV-infected A549 cells (grey bars) cells, at 1:1 and 5:1 ratios, or stimulated with PhAg (IPH1101 3 µM) as positive control. (**a**,**b**) After 18 h, culture supernatants were collected and IFN-γ (n-8) and TNF-α (n-5) were quantified by ELISA assays. Data were subtracted of the background (expanded Vδ2 T-cells alone). Data were represented as median + interquartile range.

To analyze the cytotoxic capability of Vδ2 T-cells against ZIKV infected cells, we generated Vδ2 T short-term lines from PBMC of HD. After 12 days of culture, we purified expanded γδ T-cells by magnetic separation reaching a purity >95%. We therefore co-cultured expanded Vδ2 T-cells with mock or ZIKV-infected A549 cells for 18 h at two effector/target ratio (1:1 and 5:1) and analyzed their ability to kill ZIKV-infected cells by flow cytometry using AnnexinV/PI staining. As shown in Figure 2A, the frequency of AnnexinV + -A549 cells was significantly higher in ZIKV-infected than in Mock co-cultures at both 1:1 [ZIKV-A549: median 2.4% (IQR: 1–5.7) vs. Mock-A549: 0.9% (0.0–2.4) *p* < 0.002)] and 5:1, (ZIKV-A549: 3.3 (1.7–28) vs. Mock-A549: 1.1% (0.8–3.2), *p* < 0.03) ratios. In the same way (Figure 2B), a significant higher frequency of PI + -A549 cells was observed in ZIKV-infected than in Mock co-cultures at both 1:1, [ZIKV-A549: median 6.9% (IQR: 5.5–12.1) vs. Mock-A549: 1.7% (0.0–2.7) *p* < 0.002)], and 5:1 [(ZIKV-A549: 10.4% (5.6–17.2) vs. Mock-A549: 3.9% (1.1–5.4), *p* < 0.03)] ratios indicating that expanded Vδ2 T-cells were able to kill ZIKV-infected cells. In the same experiments, we analyzed the expression of CD107a degranulation marker on Vδ2 T-cells (Figure 2C). A significant increase of CD107a+ Vδ2 T-cells was observed after co-culture with ZIKV-infected cells if compared to mock-infected cells (5:1 ratio), indicating that expanded Vδ2 T-cells degranulated after recognition of ZIKV-infected cells. Accordingly, a higher perforin release was observed in ZIKV-infected cells co-culture when compared with mock co-culture at both ratios (*p* < 0.05; Figure 2D). In contrast, no significant release of IFN-γ and TNF-α was observed (Figure 3A–D).

### 3.3. ZIKV Infection Increased NKG2D Ligand Expression in A549 Cells

**Figure 4:**
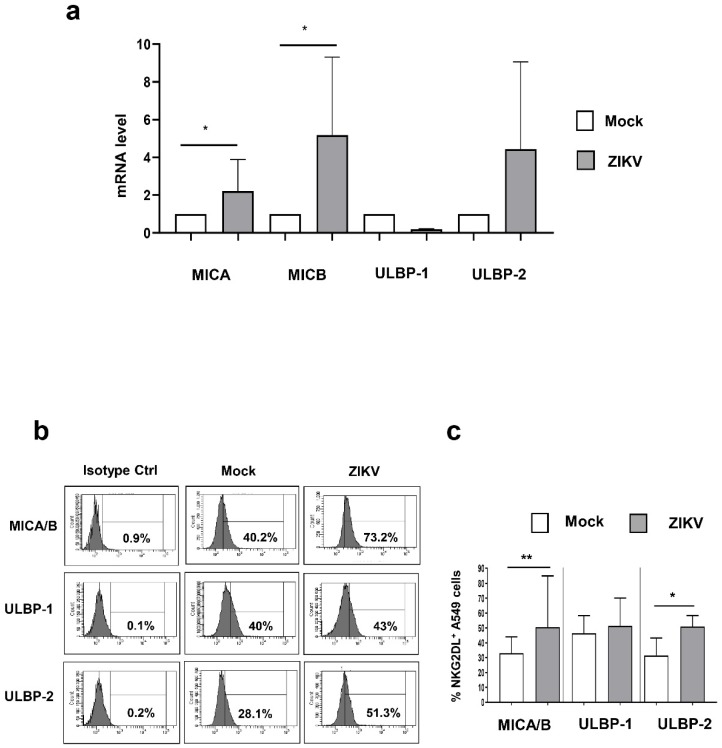
ZIKV up-modulated NKG2DLs in A549 cells. (**a**–**c**) Expression of NKG2DLs was analyzed in A549 cells infected or not for 48 h with ZIKV (MOI 1). (**a**) Relative NKG2DL mRNA levels in ZIKV-infected cells compared with control mock cells were measured by Real-time qPCR (*n*-5). (**b**,**c**) The frequency of MICA/B +, ULBP-1 +, and ULBP-2+ was measured on mock/ZIKV-infected cells. (**b**) Histograms show MICA/B, ULBP-1, and ULBP-2 expression in a representative experiment. (**c**) Median ± Range of NKG2DL+ cells were calculated in 8 independent experiments. Data were represented as median + interquartile range. * *p* < 0.05; ** *p* < 0.01.

To clarify the mechanisms involved in the recognition of ZIKV-infected cells by Vδ2 T-cells, we investigate whether ZIKV can modify the cell-surface expression of ligands for the activating NKG2D receptor expressed on Vδ2 T-cells, specifically MICA, MICB, ULBP-1 and ULBP2 molecules. The mRNA levels of these NKG2DLs were quantified by RT-PCR 48 h post ZIKV infection. Results showed a significant increase of MICA (*p* < 0.05) and MICB (*p* < 0.05) mRNA in ZIKV-infected A549 cells when compared to mock-A549 cells (Figure 4A). We observed a trend in the up-regulation of ULBP2 in ZIKV infected cells that did not reach the statistical significance (*p* = 0.125) (Figure 4A). Nevertheless, this mild mRNA up-regulation was clearly confirmed at protein level (Figure 4C). Finally, we found a trend in the down modulation of ULBP1 mRNA (*p* = 0.250) by ZIKV infection (Figure 4A) that was not confirmed at protein level (Figure 4B,C).

### 3.4. Vδ2 T-cells Kill ZIKV-Infected A549 Cells Trough NKG2D 

**Figure 5:**
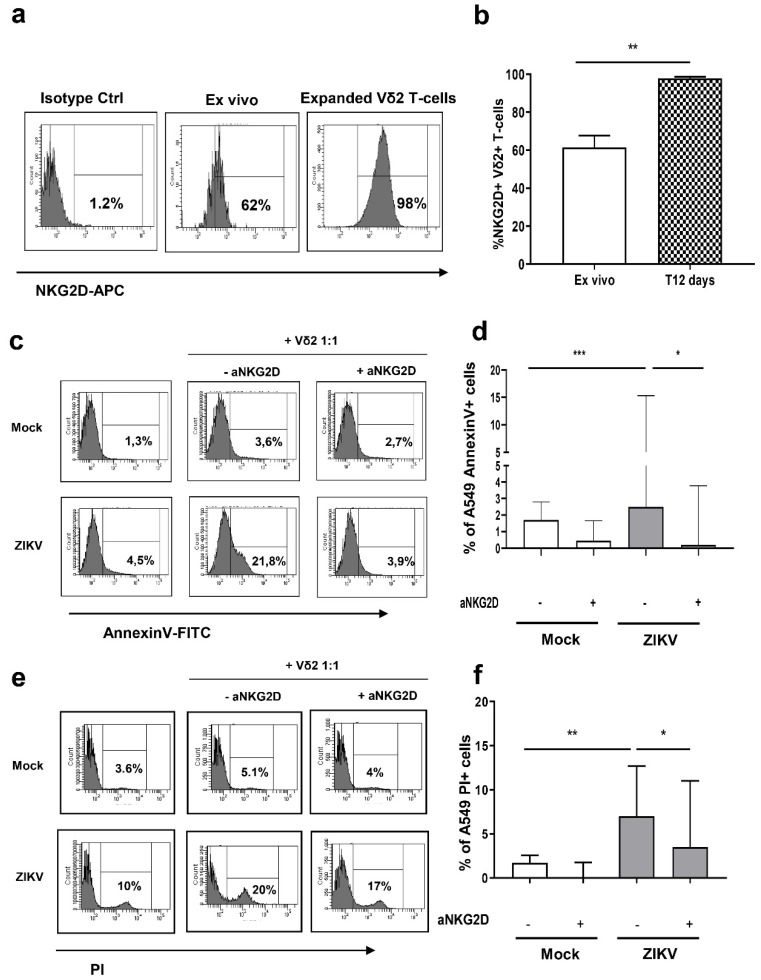
ZIKV sensitizes A549 cells to NKG2D-mediated killing by Vδ2 T-cells. (**a**) Flow cytometric panels showing the expression of NKG2D receptor on Vδ2 T-cells *ex vivo* and after 12 days of PhAg + IL-2 stimulation (Vδ2 T line) in a representative experiment. (**b**) The frequency of NKG2D+ cells among Vδ2 T-cells was compared in 9 experiments at T0 (white bars) and T12 days (squares bars). (**c**) Flow cytometry panels showing the expression of AnnexinV on mock/ZIKV-infected A549 cells after co-culture at E:T ratio of 1:1 with Vδ2 T-cells pre-treated or without blocking a-NKG2D antibody. (**d**) Comparative analysis of 9 independent experiments as the one depicted in panel C is shown. (**e**) Flow cytometry panels showing the expression of PI on mock/ZIKV-infected A549 cells after co-culture at E:T ratio of 1:1 with Vδ2 T-cells pre-treated or without blocking a-NKG2D antibody. (**f**) Comparative analysis of 8 independent experiments as the one depicted in panel E is shown. In all experiments, background (mock/ZIKV+ apoptotic/necrotic cells without expanded Vδ2 T-cells) was subtracted to all conditions. Data were represented as median + interquartile range. * *p* < 0.05; ** *p* < 0.01; *** *p* < 0.001.

NKG2D triggering by its ligands expressed on target cells transduces a potent activation signal. In order to define the involvement of NKG2D in recognition and killing of ZIKV-infected cells, we firstly analyzed the expression of NKG2D on expanded Vδ2 T-cells. Representative histograms of NKG2D expression are shown in Figure 5A. We found that a large fraction of freshly isolated ex vivo Vδ2 T-cells express NKG2D [median + IQR: 61.5% (48.9–67.6)] that was significantly increased in expanded Vδ2 T-cells [97.9% (82.0–98.7)] (Figure 5B). To verify whether Vδ2 T-cells were able to kill ZIKV-infected cells through NKG2D receptor, we pre-treated Vδ2 T-cells with anti-NKG2D (aNKG2D) neutralizing antibody. Then, we cultured untreated/treated expanded Vδ2 T-cells with mock or ZIKV-infected A549 cells for 18 h and analyzed the frequency of apoptotic cells by flow cytometry. Representative histograms are shown (Figure 5C,E). While untreated- expanded Vδ2 T-cells were able to kill ZIKV-infected cells, pre-treatment of expanded Vδ2 T-cells with aNKG2D antibody completely abrogated the cytotoxic effect (Figure 5D,F) (*p* < 0.001), indicating that Vδ2 T-cells killing of infected cells is mediated by NKG2D activation. 

## 4. Discussion

Zika virus (ZIKV) is a mosquito-transmitted flavivirus, recently emerged as a public health problem for its potential to generate epidemics and to cause congenital disease. In this context, there has been increasing interest to find strategies aimed to contain the spread of the virus globally, and to identify markers of protection/severity. 

In the years after the last ZIKV epidemic (Brazil, 2016–2017), several studies focused on the anti-ZIKV T-cell response [31] in order to depict the cell-mediated immune response and its association with complications or with humoral effective immunity. Nevertheless, the involvement of γδ T-cells in the immune response against ZIKV infection has been suggested [21] but not further characterized. Therefore, we investigated the anti-ZIKV activity mediated by Vδ2 T-cells in order to evaluate their possible role in the context of protective immunity.

We found that ZIKV-infected cells were able to induce Vδ2 T-cell expansion, confirming what observed in vivo in ZIKV-infected subjects [21]. Indeed, in ZIKV-infected patients we reported a consistent in vivo Vδ2 T-cells specific expansion during the first day’s post-infection, coming back quickly to baseline after ZIKV resolution [21]. A similar expansion of Vδ2 T-cells has been also reported during SARS-CoV infection, that persists several months after resolution and correlates with anti-SARS IgG titre [32], suggesting a role in protective immune response orchestration.

Vδ2 T-cells can exert antiviral activity against several viruses [22] through both cytolitic and non-cytolitic mechanisms. During acute ZIKV infection, expanded Vδ2 T-cells were previously shown to produce IFN-γ and express granzyme B, suggesting a potential cytotoxic capability in the first phases of ZIKV infection [21]. In the present work, we found that Vδ2 T-cells are able to recognize and kill ZIKV-infected cells through the release of perforin, confirming data obtained with other flavivirus [28]. Accordingly, we found a significant CD107a up-regulation on Vδ2 T-cells when cultured with ZIKV-infected cells.

Innate immune cells are tuned finely by the modulation of several NK receptors [33]. In particular, NKG2D is a potent activating receptor that recognizes ligands belonging to the MIC and ULBP protein families induced on target cells exposed to stressors such as viral infection, DNA damage, and oncological transformation [27]. NKG2D-expressing NK or γδ T-cells, upon recognition of the cognate ligands on infected cells, efficiently kill them through perforin/granzyme pathway [34,35,36,37].

We found that Vδ2 T-cells express high level of NKG2D and, on the other hand, ZIKV infection induced an up-regulation of MICA/B and ULBP2 on target cells, making them more susceptible to NKG2D-mediated lysis. These data are in accord with results showing an increase of stress ligands on cells infected with another Flavivirus, the West Nile Virus (WNV) [38] but in contrast with Glasner and colleagues showing an inhibition of NK cells response [39]. This discrepancy may be due to different experimental condition (different ZIKV strain, different MOI, different time kinetic analysis). In our settings, we therefore demonstrated the role of NKG2D in ZIKV antiviral activity of Vδ2 T-cells, as the neutralization of NKG2D abrogated the Vδ2 T-cells-induced infected cells killing. Moreover, a reduction of NKG2D expression on NK cells in WNV-infected patients that can act as a possible viral escape mechanism to avoid NK-mediated recognition has been reported [38]. In this context, further analysis in ZIKV-infected patients are mandatory in order to verify the effect of ZIKV infection on NKG2D expression on Vδ2 T-cells *in vivo*. 

We further quantified pro-inflammatory cytokines, such as IFN-γ and TNF-α in the supernatant co-cultures, observing no difference between ZIKV-infected and not infected cells, indicating that the Vδ2 T-cells NKG2D-mediated cytotoxicity play a dominant role in the response to ZIKV-infected cells. Several studies reported the ability of Vδ2 T-cells to produce cytokines through NKG2D stimulation (i.e., similarly to NK cells) [40,41,42,43], however, to our knowledge, data on the NKG2D-mediated cytokines production by Vδ2 T-cells activated by virus infected cells was never demonstrated. 

Results presented in this paper have been obtained *in vitro*, using expanded Vδ2 T-cells from healthy donors. The analysis of the antiviral activity of freshly isolated Vδ2 T-cells from acute and convalescent ZIKV-infected subjects may be helpful to better define their role in the protection/pathogenesis of ZIKV infection. Finally, further studies are mandatory in order to identify possible associations between Vδ2 T-cell functional activity and the wide range of clinical presentations.

## 5. Conclusions

Our data indicate that Vδ2 T-cells exert a potent NKG2D-mediated cytolytic function against ZIKV infected cells, opening questions on the role of Vδ2 T-cells in fighting ZIKV infection in other tissues, and whether the lack of Vδ2 T-cells expansion might be associated with disease complications
